# Epidemiology of self-medication with modern medicines among health care professionals in Nekemte town, western Ethiopia

**DOI:** 10.1186/s13104-017-2865-5

**Published:** 2017-10-30

**Authors:** Edao Sado, Endashaw Kassahun, Getu Bayisa, Mohammed Gebre, Ayana Tadesse, Balisa Mosisa

**Affiliations:** 1 Pharmacoepidemiology and Social Pharmacy Unit, Department of Pharmacy, College of Health Sciences, Wollega University, P.O.Box 395, Nekemte, Ethiopia; 2 Boru Meda Hospital, Amhara National Regional State, Dessie, Ethiopia; 3 Clinical Pharmacy Unit, Department of Pharmacy, College Health of Sciences, Wollega University, Nekemte, Ethiopia; 4 Pharmaceutics Unit, Department of Pharmacy, College Health of Sciences, Wollega University, Nekemte, Ethiopia

**Keywords:** Self-medication, Modern medicines, Ethiopia

## Abstract

**Objective:**

Self-medication is defined as use of medicines to treat self-recognized illnesses. It is widely used in Ethiopia. However, its extent of use is unknown among health professionals. This study aimed to assess prevalence and reasons of self-medication with modern medicines among health professionals. A cross-sectional study was conducted on the health professionals, working in the public health facilities. Data were collected from March to May, 2016 using semi-structured questionnaire. Data were entered and analyzed using statistical package for the social sciences. A chi square test was used as test of significance at 95% of confidence interval.

**Results:**

A total of 154 health professionals were enrolled, with 53% were being females. The finding revealed that prevalence of self-medication with modern medicines was 67.5%. Financial constraints (32.5%) and familiarity with medicines (24%) were the major reasons of self-medication. It also showed that self-medication with modern medicines was significantly associated with marital status (χ^2^ = 19.57, P = 0.00). Analgesics (53%) and antibiotics (36%) were the most commonly used categories of medicines. Self-medication with modern medicines was highly practiced among health professionals. Financial constraints and familiarity with medicines were the two major reasons of practicing.

## Introduction

Self-medication is defined as use of medicines by individuals to treat self recognized illnesses [1]. It includes diagnosing and treating one’s own illness and prescribing for one’s self [2, 3]. It is highly practiced in developing countries, which might be due to availability of medicines from informal sectors such as open marker, supermarket, and inadequate health care services [4]. Self-medication with both over the counter and prescription only medicines are common in developing countries [5].

Health professionals are familiar with medicines so they are different from the other population in terms of medicine use [6]. Knowledge and access to prescription medicines are potential factors for self-prescribing among health professionals. Other factors that are often quoted include the complaint of extensive demands on their time, issues of privacy and confidentiality [6]. Health professionals expect that their patients to seek appropriate health care when they get sick. However, they do not seek appropriate health care by themselves. For example, they might be inappropriately self-medicate with prescription only medicines, which lead to irrational use of medicines [7, 8]. This might be due to a perception that self-prescribing with prescription only medicines such as narcotics analgesic is safe; which is not allowed even illegal for self-prescribing [8, 9]. Various studies showed that they self-medicate with modern medicines without consulting other health care professionals, which lead to development of pill for every ill culture in the medical community. Self-medication with modern medicines has many problems. The major problems are over prescribing, misuse, use of unnecessary expensive drugs and overuse of antibiotics. These problems are great issues of global concern [10, 11].

A study conducted in India showed that a lower degree of illness is the main reason of practicing self-medication among health professionals [12] while studies done in Malaysia and Pakistan showed familiarity with treatment as a main reason of practicing self-medication [7, 13, 14].

Though different studies have been conducted in different parts of Ethiopia, the extent of self-medication among health professionals is not assessed yet. Therefore, this study was conducted to assess prevalence of self-medication with modern medicines among health care professionals; and to identify major reasons of self-medicating.

## Main text

### Methods

#### Study setting and study design

The study was conducted on health care professionals, who were working in the public health care facilities found in a Nekemte town, western part of Ethiopia. The town is located at 328 km away from Addis Ababa, the capital city of the country. The town was selected as it is the largest and most populous town in the western part of Ethiopia. At the time of data collection, there were two public health centers and one public hospital. We included all of them in the study.

A cross-sectional study was conducted from March to May, 2016 among health professionals to assess the prevalence of self-medication with modern medicines; and to identify major factors leading to self-medication with modern medicines.

#### Sample size and sampling technique

Sample size was calculated by using single proportion formula [15], which is n = z^2^ pq/d^2^, where z is estimated at 1.96 for a 95% confidence level, P is 50% of the estimated prevalence and d is the level of acceptable error estimated at 5%. After considering sample size adjustment to the population and taking 16% compensation for non-response, the sample size was determined to be 169.

We used simple random sampling to select a health professional among those working in the public health facilities found in the town. A health professional was randomly selected from the total health professionals working in the public health care facilities. He/she was included in the study if he/she was available in the health facilities during data collection.

#### Data collection tool

Self-administered semi-structured questionnaires were adapted from previously published article [13]. The questionnaire was translated into Afan Oromo, the official language of the study zone by a panel of experts fluent in the language. The Afan Oromo language version was used to collect data. Questionnaire was pre-tested on the 10% of studied health care professionals, who are working in the public health care facilities found in a Ghimbi town. Data collection was commenced after small amendment was made on the questionnaire in the field of professionals based on the results of pretest.

#### Data management and analysis

Data were coded, entered and analyzed using Statistical Package for the Social Sciences (SPSS) version 17 for windows, and Microsoft Office Excel 2007. Descriptive statistics were expressed by using frequencies and proportions. A Chi square was used as test of significance at 95% of confidence interval. A P value of 0.05 or less than 0.05 was used as the cut-off level for statistical significance.

#### Ethical clearance

Ethical clearance was obtained from the Institutional Research Ethics Review Committee of Wollega University, College of Health Sciences. A letter of cooperation was written from the Department of Pharmacy to the concerned health facilities for further cooperation. The objective of the study was explained to the study participants and a written consent was obtained from each participant.

### Results

#### Socio demographic characteristics of respondents

A total of 169 health professionals were recruited but only 154 (91.1%) of them completed questionnaire. Among 154 respondents, 81 (53%) were females. Seventy-six (49%) of the respondents were in the 20–29 age groups and 54 (31.5%) were in the 39–39 age groups while the rest 24 (15.6%) were aged more than 39 years. Sixty-two (40%) respondents had been working as health care professionals for 5 or less than 5 years. One hundred five (68.2%) of the respondents were unmarried. Majority 88 (57.2%) of the respondents were nursing professionals (Table 1).

**Table 1:** Socio demographic characteristics of respondents

Variables	Number (n = 154)	Percentage
Gender		
Male	73	47
Female	81	53
Age		
20–29	76	49
30–39	54	31.5
> 39	24	15.6
Years of practice		
≤ 5	62	40
5–10	56	37
≥ 10	36	23
Marital status		
Unmarried	105	68.2
Married	49	31.8
Field of practice		
Medical	13	8.4
Nurses	88	57.1
Pharmacy	13	8.4
Other^a^	40	26

Other^a^: Includes laboratory personnel, health officer, sanitarians, and anesthetics

#### Reasons for self-medication and category of medicines used

Among 154 respondents, 104 (67.5%) of them were practicing self-medication with modern drugs within last 2 months of recall period. Analgesics 56 (36.1%) and antibiotics 37 (23.9%) were the most commonly used medicines categories. Financial constraints 50 (32.5%) and familiarity with medicines 37 (24%) were the most commonly mentioned reasons to self-medicate with modern medicines (Table 2).

**Table 2:** Reasons for self-medication and category of used medicines

Variables	Frequency	Percentage
Reasons for self-medication		
Familiarity with drugs	37	24
Mildness of illness	21	13.6
Privacy	26	16.9
Less cost/financial constraint	50	32.5
Lack of time	11	7.1
Others	9	5.9
Category of medicines used		
Analgesics	56	36.1
Antibiotics	37	23.9
Oral contraceptives	26	16.8
Antacid	20	12.9
Oral hypoglycemic agents	10	6.5
Others	6	3.9

#### Factors influencing self-medication with modern drugs

To assess the association of self-medication with socio-demographic variables of studied participants, we calculated Chi square test. We found that self-medication was significantly associated with marital status (χ^2^ = 19.57, P = 0.00) (Table 3) at 95% of confidence interval.

**Table 3:** Factors influencing self-medication with modern drugs

Variables	Self-medication with MDs		Chi square
	Yes (n = 104)	No	
Gender			
Male	49	24	χ^2^ = 0.2312
Female	55	26	P = 0.6306
Age			
20–29	56	20	χ^2^ = 2.77
30–39	34	20	P = 0.2503
≥ 40	14	10	
Year of experience			
≤ 5	46	16	χ^2^ = 2.1787
5–10	36	20	P = 0.3364
≥ 10	22	14	
Marital status			
Unmarried	85	20	χ^2^ = 19.57
Married	19	30	P = 0.0000
Field of professionals			
Medical	13	0	–
Nurses	56	32	
Pharmacy	10	3	
Other^a^	25	15	

^a^Laboratory personnel, Health officer and Sanitarians

### Discussion

The aim of the present study was to assess epidemiology of self-medication with modern medicines among health care professionals. The finding of this study suggested that two-thirds (67.5%) of health professionals were self-medicating with modern medicines. This figure was comparable with findings reported from study conducted in Malaysia [13]; and it was less than the findings reported from study conducted in India, Pakistan and Ghana [10, 12, 14]; and it was higher than the reports from study done in America [16]. This discrepancy might be due to different factors such as variation in income of the professionals, availability of social health insurance for professionals, and governing laws, which prohibit sales of prescription only medicines as over the counter.

In contrary to study conducted in India, which showed self-medication was significantly associated with female sex, it was not significantly associated with sex of the studied populations in this study [12]. However, it was significantly associated with marital status of the studied population (X^2^ = 19.57, P = 0.00) at 95% confidence interval, where unmarried health professionals had more tendency to self-medicate with modern medicines. This significance might be due to the number of surveyed respondents as majorities were unmarried, which might be outlier the finding. Although self-medication was not significantly associated with age, the prevalence of self-medication higher in the respondents with lower age categories. This was in contrary to the reports of study conducted by Boateng, which showed higher rate of self-medication with higher respondent age group [10].

This study revealed that financial constraints and familiarity of the medicine were major reasons of practicing self-medication. This finding was similar to report from study done in Malaysia [13]. However, busy life schedule and previous experience of medicines were reported as the major factors in the study conducted in Pakistan [14]. Different studies also showed that low severity of illnesses was the main cause for the practice of self-medication, unlike to this finding [17, 18].

The study also revealed that analgesics and antibiotics were the most commonly used categories of medicines. However, this finding was inconsistence with the reports from studies done by Boateng and Tensaw, which reported a higher rate of antibiotics and analgesics [1, 10]. This high prevalence of analgesic for self-medication might be associated with their availability as over the counter. It also suggested that antibiotics were also available as over the counter medicines, where their uses were not supported by laboratory investigation. Antibiotics are susceptible to the risk of misuse and yet they are often exposed to the high rate of self-medication practices [19, 20]. Use of antibiotics as over the counter drugs may cause the development of bacterial resistance.

### Conclusions

The aim of this study was to assess prevalence of self-medication with modern medicines among health care professionals. The study revealed that self-medication with modern medicines was highly practiced among health professionals. Familiarity with medicines, and financial constraints were the major mentioned reasons of self-medicating. Analgesics and antibiotics were the most commonly used categories of drugs.

Based on the findings, we proposed the following recommendations to minimize prevalence of self-medication:

- In order to overcome financial constraints, social health insurance should be implemented.
- To minimize the use of antibiotics for self-medication, sales of antibiotics as over the counter medicines should be prevented through effective supervision.

## Limitations

This study has two major limitations. First, the study was carried out among health professionals working in the public health facilities only. Thus the findings cannot be generalized to all health professionals working in Ethiopia. Second, 2 months recall period was used to collect information hence it might be subjected to recall biases. However, to our knowledge this article was the first to assess prevalence of self-medication with modern medicines among health care professionals in Ethiopia.

## References

[1] Tensaw A (2004). Self-medication practice in Addis Ababa. A prospective study. Ethiopian J Health Sci.

[2] Montastruc JL, Bagheri H, Geraud T, Lapeyre MM (1997). Pharmacovigilance of self-medication. Therapy.

[3] Abay SM, Amelo W (2010). Assessment of self-medication practices among medical, pharmacy, and health sciences students in Gondar University, Ethiopia. J Young Pharm.

[4] Shankar PR, Partha P, Shenoy N (2002). Self medication and non doctor prescription practices in Pokhara valley, western Nepal, a questionnaire based study. BMC Farm Pract.

[5] Murray MD, Callahan CM (2003). Improving medication use for older adults an integrated research Agenda. Ann Intern Drug.

[6] Rosen Ilene M, Christie Jason D, Bellini Lisa M (2000). Mentally ill doctors. Br J Hosp Drug.

[7] Montgomery AJ, Bradley C, Rochfort A, Panagopoulou E (2011). A review of self-medication in physicians and medical students. Occup Med.

[8] University of Florida. The legality and ethics of self-prescribing. Drugs Ther Bull. 2006;20(7).

[9] Council Medical (2009). Guide to professional conduct and ethics for registered medical practitioners.

[10] Boateng DP. Self-medication among doctors and pharmacists at the Korle Bu Teaching Hospital: Department of Clinical and Social Pharmacy, Kwame Nkrumah University of Science and Technology. 2009.

[11] Solomon W, Nbebe G (2003). Practice of self-medication in Jimma town, Ethiopia. J Health Dev.

[12] Gholap MC, Mohite VR (2013). Assess the self-medication practices among staff nurses, Krishna Institute of Nursing Sciences, Karad, India. Indian J Sci Res.

[13] Ali AN, Tiong J, Kai K, Keat CC, Dhanaraj SA (2012). Self-medication practices among health care professionals in a Private University, Malaysia. Int Curr Pharm J.

[14] Shoaib MH, Yousuf RI, Anjum F, Saeed L, Ghayas S, Ali T (2013). Survey based study on the use of non-prescription drugs among pharmacists and non-pharmacists, University of Karachi, Pakistan. Afr J Pharm Pharmacol.

[15] World Health Organization. Sampling methods and sample size. In: Omi S, editor. Health research methodology. A guide for training in research methods. 2nd ed. 2001.

[16] Christie JD, Rosen IM, Bellini LM, Inlesby T, Lindsey J, Aper A (1998). Prescription drug use and self-prescription among resident physicians. J Am med Assoc.

[17] Caamano F, Fgueiras A, Lado Lema E, Gestalo-Otero JJ (2000). Self-medication: concept and “User” profile. Gac Santi.

[18] Auta A, Omale S, Folorunsho TJ, David S, Banwat S (2012). Drug vendors: self-medication practices and drug knowledge. N Am J Med Sci.

[19] Gutema GB, Gadisa DA, Kidanemariam ZA, Berhe DF, Berhe AH, Hadera MG (2011). Self-medication practices among health sciences students: the case of Mekelle University. J Appl Pharm Sci.

[20] Davidson S, Schattner P (2003). Doctors’ health-seeking behavior. A questionnaire survey. Med J Am.

